# Injuries among children and adolescents in a rapidly growing urban African metropolis: a cross-sectional survey of 1,968 households in Dar es Salaam, Tanzania

**DOI:** 10.7717/peerj.10048

**Published:** 2020-10-15

**Authors:** Mónica Alejandra Pérez Méndez, Hamisi A. Kigwangalla, Till Bärnighausen, Michael Lowery Wilson

**Affiliations:** 1 Heidelberg Institute of Global Health, University of Heidelberg, Ruprecht-Karls-Universität Heidelberg, Heidelberg, Germany; 2 Centre for Injury Prevention and Community Safety (CIPCS), PeerCorps Trust Fund, Dar es Salaam, Tanzania

**Keywords:** Child and adolescent health, Environmental health, Care-giving, Epidemiology, Global health

## Abstract

**Objectives:**

To assess the patterns and incidence of child and adolescent injury and explore associations with household deprivation and child characteristics in a low-income urban setting.

**Study Design:**

Cross-sectional household survey in Dar es Salaam, Tanzania.

**Methods:**

Data collection took place during July 2009. Injuries requiring medical attention were recorded with a one month period of recall. A total of 1,968 households representing 3,927 children and adolescents were visited by health workers. Gender-, age-, and type-specific injury incidence was compiled. Odds ratios were calculated to measure associations with child injury, perceived deprivation, household characteristics and child characteristics.

**Results:**

One household in five reported injuries. The estimated incidence was 3.2 per 10,000 child-years. The most common identifiable injuries were falls (41%), cuts (22%) and burns (16%). Male and younger children aged 1–4 years were at higher risk (respectively OR = 1.36; *p* = 0.004; OR = 1.47; *p* ≤ 0.001).

**Conclusions:**

In Dar e Salaam injuries are common. Future investigations should take into account both subjective and objective measurements of relative household deprivation and a clear criteria for the assessment of injury severity in community-based survey contexts.

## Introduction

Injuries are a leading cause of both disability and mortality for children ages 0-18 worldwide (Kendrick et al., 2005; Roberts, DiGuiseppi & Ward, 1998; Towner & Towner, 2009), accounting for nearly 4.48 million deaths in 2017 (Roth et al., 2018). Approximately 90% of these injuries are unintentional and occur in mostly low- and middle-income countries (LMICs) (Bartlett, 2002). In sub Saharan Africa (SSA), injuries have now become the third leading cause of death (Moshiro et al., 2001; Peden et al., 2008) and the region has the world’s highest rate of unintentional injury deaths among children in particular aged 1-4 years at 100.5 per 100,000 (Khan et al., 2015). In addition to these deaths, many more children are left with some form of disability or disfigurement that impedes their full integration in social end economic life (Peden et al., 2008).

Young children are especially vulnerable to injuries that have life threatening outcomes (Bartlett, 2002). Their physiological characteristics, such as higher head to body weight ratio, makes them a particularly at risk for falls that increase the likelihood of head injuries (Wilson, 2012). Adolescents, increasingly take on responsibilities in the home such as caring for their younger siblings and assisting with household duties such as cooking meals - often without adequate supervision (Peden et al., 2008). In spite of this, injuries among children and adolescents remain a largely neglected public health problem, particularly in resource limited settings, where vulnerability is exacerbated (O’Campo et al., 2000).

Few countries in the SSA region have population-based data on injuries (Ruiz-Casares, 2009). Those which exist suggest that falls and burns are the main types of injuries among children (Forjuoh, 2003; Forjuoh & Li, 1996; Kobusingye, Guwatudde & Lett, 2001; Van Niekerk, Rode & Laflamme, 2004; Gyedu et al., 2015). In Tanzania, two studies using population derived data document injuries as an important source of morbidity (Moshiro et al., 2005a) and mortality (Moshiro et al., 2001) but provide incomplete data on child injury. Other Tanzanian studies rely mainly on health records (Justin-Temu et al., 2008; Kahabuka & Mugonzibwa, 2009; Mselle, 1998) which may underestimate the problem (Bartlett, 2002). Besides problems in coverage, health records provide little information on potential risk factors (Kahabuka & Mugonzibwa, 2009; Mselle, 1998).

Apart from age and sex differences in injury risk (Dowd, Keenan & Bratton, 2002; Ruiz-Casares, 2009), material deprivation has also been found to be an important risk factor (Laflamme, Hasselberg & Burrows, 2010; Ma et al., 2019). Additional factors include: large family size (Bang, Ebrahim & Sharma, 1997; Janson et al., 1994; Rahman, 2005; Sharma et al., 2018), children being taken care of by older siblings (Forjuoh & Li, 1996; Rahman, 2005), health and well-being of caregivers being compromised (Bang, Ebrahim & Sharma, 1997; Janson et al., 1994) and the education levels of caregivers (Bangdiwala et al., 1990; Ma et al., 2019).

This aim of the present study was to investigate the patterns incidence of injuries among children and adolescents in Dar es Salaam (DES), Tanzania. It also explores associations between several child and household characteristics.

## Methods

### Setting

DES is Tanzania’s largest city and most important economic center. At the time of the study DES had a population of approximately 2.5 million and about 600,000 households. Roughly 33% of the DES population was between 0–14 years of age (National Bureau of Statistics, 2002; National Bureau of Statistics, 2003). The city is divided administratively into three municipalities and 74 electoral districts called wards (National Bureau of Statistics, 2003).

### Sampling

**Figure 1:**
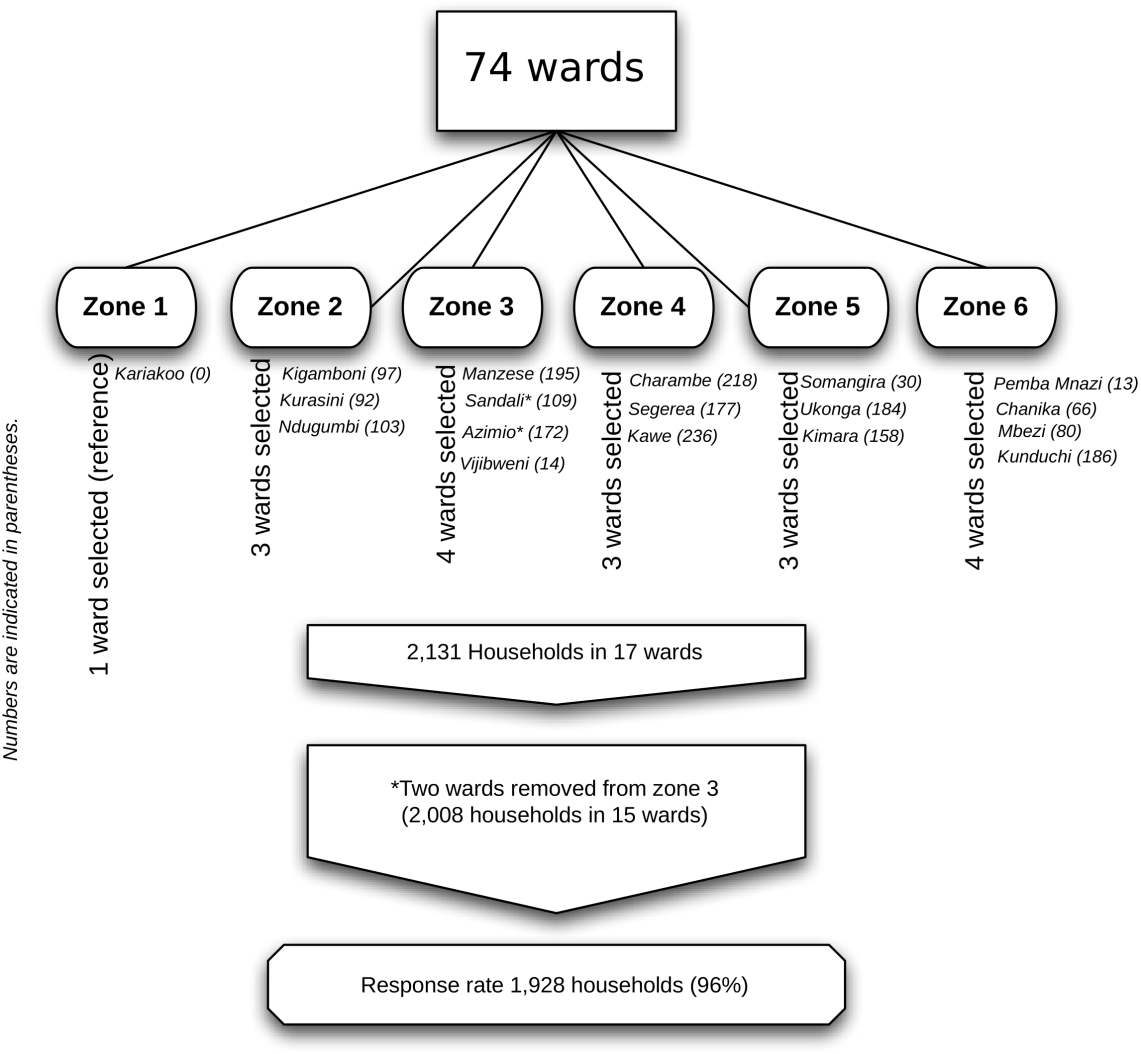
Sampling strategy and sampled wards.

A multi-stage sampling process was employed. We assumed a 5% incidence of all cause child injury based on prior estimates in the region (Moshiro et al., 2005a). The sample size with 80% power and 0.05 precision was calculated at 1,968 households with at least one child under age 19. The operational definition for a household was “the place where a caregiver and the child/children under their care regularly meet and share meals”. Sample size calculations were carried out using StataSE version 10.1. Each ward was allocated into one of six zones using ArcGIS version 9.2. Zones were determined based on ward population densities and distance from the central business district. A computer-assisted random sampling of two or three wards (depending on population density) by zone was then conducted, resulting in 17 selected wards. Within selected wards, a proportional sample of households was selected for visitation (1.1%) which yielded 2,131 households. Two wards (Vijibweni and Sandali) were removed to reduce over-sampling in areas that were demographically similar. An overview of the sampling procedure and sampled wards is provided in Fig. 1.

### Strategy and instrument for data collection

The first household to be visited in each ward was selected at random. Face-to-face interviews were conducted by trained and experienced interviewers. The family member with most responsibility for the care of the children in the home was asked to be the participant. Each introduction was accompanied by information regarding the purpose of the study and the confidentiality of the responses. The interview was conducted if the caregiver provided their verbal consent to participate in the survey. Once the interview was completed, interviewers visited neighboring households. If an interview could not be conducted during the first visit, the household was revisited once. There were 32 households which declined to be interviewed, homes of the research team members were avoided (*n* = 1) and those which could not be reached due to large gates or similar environmental barriers (*n* = 47) were excluded (a total of 80 households).

The data were collected using a modified version of the Global Childhood Unintentional Injury Surveillance (GCUIS) instrument (Hyder et al., 2009). GCUIS, designed to collect injury data in emergency, was converted to one suitable for use in a community-based setting. The survey contained three parts. In parts A and C, 15 questions collected demographic and household information such as age, gender and occupation of caregivers, the number of rooms in the home, types of cooking fuels used, whether caregivers had suffered from any physical impairment or restriction of movement within the reference period (one month), perceived deprivation (if they were able to meet their daily needs), and number of people residing in the home.

Part B collected information on injuries occurring in the home among children within the reference period: the number of children under the care of each caregiver, their ages and gender, the location of the injury event, who provided first aid, area of the body affected, type of injury, activity leading to injury, illnesses suffered during the reference period, broken bones as a result of the injury, whether the injury was intentional or unintentional, perceived long term effect of the injury, and other injuries occurring within the reference period.

For injury types we utilized the World Health Organization (WHO) guidelines for injury surveillance (Holder, Peden & Krug, 2001). Of the 12 types of injury defined by the WHO, we retained 6 covering injuries occurring in the home: falls; burns; cuts; poisoning; animal/insect bite and injury intent with others being classified as injuries of unspecified nature. The operational definition of injury for the purposes of the study was: *“an event occurring in or near the home within the preceding month that resulted in a poisoning, animal/insect bite, fall, burn, cut, or other harm whether intentional or unintentional requiring any form of treatment”*. Proxy measurements for injury severity were used. Caregivers were asked whether the injury resulted in a broken bone, indicating emergency treatment and whether injury care required caregivers to borrow funds to access treatment. No other clinical measures of severity were used.

The questionnaire was originally drafted in English, translated into Swahili, back translated and then pilot tested in the field. Pilot testing was carried out during two days among 50 households selected from two communities 2 km apart in the ward of Ilala Boma in Dar es Salaam. Completed questionnaires were checked while in the field for completeness and accuracy by two field supervisors. Participant responses were entered into a spreadsheet for data sorting, checking and cleaning by two data entry persons.

### Data treatment and analysis

In compiling the injury incidence, numerator data (injury events) was obtained by compiling the total number of first reported injuries among each child in each home. Denominator data was obtained from the total number of reported children from each home. Perceived deprivation (poverty) was measured by asking caregivers *“Are you able to meet your daily needs for food, clothing and medicines?”* Response categories for caregiver physical impairment were: unable/difficulty walking; limited use of arms/hands; poor vision and other. Child age, gender and health status (illness present) were also asked. The age categories defined in the WHO guidelines for injury survey and surveillance were used: <1; 1–4 5–9; 10–14 and 15–18 (Holder, Peden & Krug, 2001; Kobusingye, Guwatudde & Lett, 2001).

Malaria, pneumonia, tuberculosis were considered among the major childhood illnesses reported by caregivers within the reference period. All independent variables were first entered separately into bivariate models with injury as the dependent variable. Subsequently, we carried out multivariable logistic regression analyses for each of the variables found significant in the bivariate analyses. Odds ratios are reported to depict the strength and direction of associations between the independent variables and the risk for injuryn along with 95% confidence intervals. Statistical significance for all analyses were indicated at *p* < 0.05. To account for child clustering, analyses are reported with robust standard errors (rSE). All statistical analyses were carried out using StataSE version 10.1.

### Ethical considerations

Ethical approval along with a permit to conduct research was granted by the Tanzanian Commission for Science and Technology (COSTECH) permit CST/RCA 2009/117/2009.

## Results

Between July 8–22, 2009, a total of 1,928 households participated in the study (response rate 96%), representing 3,927 children. The average number of children living in each household was 2 (SD = 1.19). Of participating households, approximately 41% were one-room dwellings. The mean number of residents per household was 4.8 (SD = 2.2). Caregivers were overwhelmingly female (93%) and roughly 52% were homemakers with 29% being self-employed. Some form of major illness was reported among 32% of the children during the reference period. A total of 416 injuries were reported among 371 households (20% of all households). The incidence rate for all injuries aggregated is 3.2 per 10,000 child years (95% CI [2.95–3.53]). Cross tabulations of injury type by age groups and sex are provided for injuries in Table 1. Children aged 1–4 years were over-represented in reported injuries near and around the home environment. In absolute terms, males were over-represented in every age category with the exception of age category 10–14.

**Table 1:** Sex and age distribution of injuries occurring in the home among children in Dar es Salaam, Tanzania for a one-month period of recall July 2009.

**Sex and age group**	**Number of children**	**Number of injuries**	**Rate of injuries (per 10,000 pop).**
Males			
<1	149	9	604.0
1–4	587	75	1,277.7
5–9	570	57	1,000
10–14	448	25	558.0
15–18	208	14	673.1
Total (males)	1,956	180	920.2
Females			
<1	160	8	500
1–4	544	60	1,102.9
5–9	590	41	753.7
10–14	452	27	597.3
15–18	214	13	607.5
Total (females)	1,960	149	760.2
Total (all children)	3,916^a^	329^a^	840.1

**Notes.**

a Missing age and corresponding injury responses on 11 children.

**Table 2:** Injury distribution and injury rate among children by cause for a one-month period of recall July 2009 in Dar es Salaam, Tanzania in 2009.

**Injury causes**	**Number of injuries**	**Injury rate (per 10,000 pop).**
	N (%)	
Falls	169 (41)	430.4
Cuts	93 (22)	236.8
Burns	67 (16)	170.6
Animal bite	2 (<1)	5.1
Poisoning	1^a^ (<1)	2.5
Intentional	6 (1.4)	15.3
Unspecified injuries	78 (18.8)	198.6
Total	(100)	1,059.3

**Notes.**

a Fatal outcome.

Injuries by type and incidence are presented in Table 2, revealing that, all ages aggregated, falls, cuts and injuries of unspecified cause were the most frequently reported.

In Table 3 ORs are reported for the child characteristics. Male children were over represented among those who experienced an injury during the recall period. Caregivers who reported a child who had an illness were 30% more likely to report having had an injury. Children under the age of five years were 47% more likely to have experienced an injury during the recall period, the highest of all the considered age categories. Being an adolescent aged 10–14 years appears to have had a protective effect.

**Table 3:** Demographic associations with child injury risk for a one month period of recall in Dar es Salaam, Tanzania, 2009.

**Variable**	**N (of 3,927)**	**aOR (rSE)**	**95% CI**	*p*-value
Age^a^ ( <1)	309	0.63 (0.143)	0.40–0.98	0.40
(1–4)	1,131	1.47 (0.160)	1.19–1.82	<0.001
(5–9)	1,162	1.13 (0.126)	0.91–1.41	0.26
(10–14)	900	0.68 (0.092)	0.52–0.88	0.004
(15–18)	417	0.80 (0.145)	0.57–1.15	0.23
Illness present	1,265	1.30 (0.134)	1.05–1.61	0.015
Male gender^b^	1,961	1.36 (0.142)	1.10–1.66	0.004


**Notes.**

a Missing age data on 8 children.

b Missing gender data on 3 children.

In Table 4 we depict the associations between selected aspects of the household environment and child injury risk. Caregiver reported household deprivation was associated with a statistically significant increase in reported injuries OR = 1.32: CI [1.04–1.66]. None of the other considered household factors was found to cross the threshold for statistical significance.

**Table 4:** Household environment and associations with child injury risk in Dar es Salaam, Tanzania, 2009.

**Household environment variable**	**Reporting yes (%)**	**OR (95% CI)**	*p*-value
Household deprivation	876/1,920 (45.6%)^a^	1.32 (1.04–1.66)	0.015
4 or more children in the home	222/1,928 (11.6%)	0.75 (0.52–1.10)	0.154
6 or more children in the home	29/1,928 (2%)	0.87 (0.33–2.30)	0.783
>5 persons living in the home	582/1,925 (30%)^b^	0.96 (0.75–1.23)	0.747
>10 persons living in the home	85/1,925 (4%)^b^	0.51 (0.20–1.29)	0.152
Family lives in a one room home	793/1915 (41.4%)^c^	1.02 (0.81–1.28)	0.869
Family lives in home with > three rooms	569/1,915 (30%)^c^	0.76 (0.16–3.45)	0.724

**Notes.**

a Data missing on eight caregivers.

b Data missing on three caregivers.

c Data missing on 13 caregivers.

Caregivers themselves were most often the first to respond (60%) in the event of an injury, with friends or other family members (21%) providing assistance where the main caregiver was not available. Within the recall period, 5% of injuries resulted in broken bones according to the interviewed caregivers. Approximately 98% of the sustained injuries were perceived by caregivers to result in less than a 6-week period of restricted normal activity. Less than 1% of injuries were perceived to cause any permanent physical impairment. One death was reported within the recall period. Approximately of 11% caregivers reported needing to borrow money to provide care for an injured child. Most injuries (74%) occurred during leisure time and were not non-sport related (5%). Activities of daily living such as bathing or cooking contributed to 12% of injuries.

## Discussion

The study demonstrates that in DES, the incidence of child injuries in and around the home is considerable, reaching 3.2 per 10,000 child years for all ages aggregated. The crude injury rate for children in DES (840 per 10,000) is higher than that of the DES population in general (245 per 10,000) (Moshiro et al., 2005b) for a lower severity threshold. This is particularly true for the 1–4 age group when compared with the other age groups. This finding is in line with previous research from Tanzania highlighting injuries as an important contributor to morbidity and mortality among the general population (Justin-Temu et al., 2008; Kahabuka & Mugonzibwa, 2009; Kimati, 1977).

The types and distribution of child injury in and near the home in DES appear to reflect those found in other SSA settings, with falls, followed by cuts and burns being higher than the global average (Bartlett, 2002; Ruiz-Casares, 2009). As expected, young age, male gender and illness were associated with excess injury risk (Ruiz-Casares, 2009). While falls, cuts and burns were the major specified sources of injury, the absolute and relative figures remain uncertain due to the high proportion of unspecified injury types. The high proportion of falls also appears to be significant when compared with the proportions of burns and cuts, a finding reflected in WHO reports of injury distribution in the region (Peden et al., 2008). There were fewer cases of poisoning otherwise reported as being important source of injury mortality near the home in the region, especially in rural areas (Peden et al., 2008; Ruiz-Casares, 2009). Poisoning has generally been associated with farming activities such as pesticide application (Mull & Kirkhorn, 2005). The same may also be true for drowning and animal attacks where household proximity to open bodies of water (Peden & McGee, 2003), levels of supervision (Ruiz-Casares, 2009) and proximity to animals (mainly dogs) expose children to these risks (Fevre et al., 2005).

Given that a variety of health issues were captured in the sample, and against indications in the literature of a possible relationship between health status of the caregiver and childhood injuries, this question was investigated in a previous publication by the authors using these data (Wilson et al., 2012). As discussed elsewhere (Ruiz-Casares, 2009), physical impairments, in particular among older caregivers, combined with household deprivation may give rise to increased injury burdens among already vulnerable families. Thus the importance of research into care arrangements may hold important implications for child safety. The ratio of children to caregivers was also an especially important consideration given a tendency towards not only larger families, but also extended care networks with in some cases, near full-time care being provided by older siblings in many African settings (Ruiz-Casares, 2009). However, the risks for injury were not increased for caregivers having 3, 5 or 6 or more children under their personal care in the present study. Conversely it was also not protective having fewer children per caregiver (two or fewer). These results suggest that there is a scope for further research and increased attention to the burden of child injury in resource limited settings. This includes further research to improve the understanding of the mechanisms of safety provision in home environments.

Although 12 households (<1%) reported more than one injury per child, in all analyses, only the first injury reported for each child was used in order to avoid the dependency effect of repeated events. In the absence of longitudinal follow-up, the potential benefit of including in analyses rates of re-injury are negligible (Hang, Bach & Byass, 2005; Hang, Byass & Svanström, 2004). Subsequent injury events, and the resultant disability associated with each, are also difficult to independently verify in the context of one cross-sectional study (Moshiro et al., 2005a; Rezapur-Shahkolai et al., 2009).

The study has a number of strengths that contribute to the accuracy of the results, a high response rate (96%), a large and diversified geographical coverage of living environments and circumstances relevant for injury in DES, and a potentially low recall bias for non-fatal injury incidence, minimized by utilizing a one-month reference period. An important source of possible bias was the lack of a comparable severity criterion. Without a severity threshold for inclusion, minor events of no medical significance are potentially over represented. In the present study we did not ask if caregivers actually needed to seek medical care outside of the home when the children suffered an injury. However, we did ask caregivers if the family had to borrow money to provide care for the injured child. In this way, we are able to make a potentially strong case for severity by way of proxy with the question of having to borrow money. Further research will have to be done to validate the utility of this as an actual severity criterion for injuries in community-based contexts, particularly where there may be an absence of clinical or insurance data to corroborate self-reported data.

Caregiver responses were biased towards younger children. Younger children likely spend more time in the home in close proximity to caregivers, and this represents a potentially increased awareness of injuries occurring among these children when compared with adolescents. The increased mobility, physical attributes and thus greater ability of adolescents to explore their environments, may mean that most injuries occurring among them may to a larger extent, occur outside of the home (Ruiz-Casares, 2009). Consequently, information on injuries among adolescents may likely be under reported in this study.

The majority of non-response was observed in areas of higher socio-economic status. It was not possible to control for this bias towards responses from those of reduced means. As a result it is not possible to conclude that this study is equally representative of all economic strata of DES. An additional source of potential bias arises from capturing very little information on injury mortality among the population due to the shortened reference period.

The lack of statistical significance found in the analysis of household crowding compared with other studies (Peden et al., 2008; Ruiz-Casares, 2009), may be due to insufficient variability among households in the sampled areas. It is likely that the absolute number of falls requiring treatment in this study has been overestimated, when compared with burns or cuts. This may be due to caregivers reporting more information rather on the fall events as opposed to fall consequences compared to what would be done by health professionals (Holder, Peden & Krug, 2001). The ability of the survey to capture accurate fall information is therefore likely compromised by poor narratives resulting from forced categories.The ability to asses the quality of construction of the household as a risk factor for falls was not possible.

While poverty indicators used in other studies have typically used quantitative measurements such as housing budget surveys (Moshiro et al., 2005a; Moshiro et al., 2005b), this study utilizes a qualitative definition underscoring the multi-dimensional nature of poverty. Thus “perceived deprivation” as measured by this study provides contextual and culture specific information derived from social comparison, stigma, social identity and perceived similarity with others of similar social standing in the community (Zagefka & Brown, 2005). However, the results of its use are difficult to generalize to other settings. Further studies could use both subjective and objective measures of deprivation for broader comparability purposes.

Finally, the proportion of injuries of unspecified nature was high enough to make the absolute and relative values for all other injury types uncertain. Despite these limitations, this study provides important information on the occurrence of injuries among children in DES, the role of household factors and child characteristics on injury risk.

## Conclusions

The injury types, gender and age distributions observed in this study are similar to those of earlier studies (Kobusingye, Guwatudde & Lett, 2001; Moshiro et al., 2001; Moshiro et al., 2005a). Greater research attention should be given to understanding the mechanisms of caregiver health may influence injury risk. Future investigations ought to examine what is needed to enhance home safety conditions in low-income contexts and to understanding injury patterns and associated morbidity among adolescents. The information from this study may prove useful in informing the design of further research examining injury mechanisms in or near the home and in LMIC settings in particular.

##  Supplemental Information

10.7717/peerj.10048/supp-1 Supplemental Information 1 Survey form and codebook

10.7717/peerj.10048/supp-2 Supplemental Information 2 The responses to the survey questions

## Additional Information and Declarations

### Data Availability

The following information was supplied regarding data availability: The raw data include all survey responses used in the analyses in the study and are available in the Supplementary Files.
