# Supplementary material for: Injuries among children and adolescents in a rapidly growing urban African metropolis: a cross-sectional survey of 1,968 households in Dar es Salaam, Tanzania
Source: PeerJ. 2020 Oct 15;8:e10048. doi: 10.7717/peerj.10048 (PMC7568856; doi:10.7717/peerj.10048)
Supplement: Supplemental Information 1 [file peerj-08-10048-s001.pdf]

Peercorps Child Injury Survey

Group:	Ward:	House No.:	Child No.:

A. Respondent Information/Taarifa ya mjibu

We would like to first ask you some questions about yourself. *Tutaanza kwa kukuuliza maswali kuhusu wewe binafsi.*

**1. What is your age? (years)/Umri__________**<br>77. Refused to answer/Alikataa kujibu<br>88. Does not know/ Hajui ☐	**2. Sex of respondent (observed) /Jinsia**<br>1. Female/mke<br>2. Male/mme ☐
**3. What is your relationship to the children in the household?/Unauhusiano gani na watoto unaowalea?**<br>1. Biological Mother/mama mzazi<br>2. Stepmother/mama wa kambo<br>3. Family member (female)/ndugu wa kike<br>4. Family member (male)/ndugu wa kiume<br>5. Other/nyingine____________<br>77. Refused to answer/Alikataa kujibu<br>88. Does not know/ Hajui<br>☐	**4. What is the highest level of education that you have completed? /umefikia kiwango gani cha elimu?**<br>1. Never went to school/sikusoma<br>2. Primary school education/elimu ya msingi<br>3. Incomplete primary /Sikumaliza shule ya msingi<br>4. Secondary school education/elimu ya sekondari<br>5. Incomplete secondary / Sikumaliza shule ya sekondari<br>6. Advanced secondary school education/elimu ya juu ya sekondari.<br>7. Attended university/ elimu ya chuo kikuu<br>8. Other/nyingine_______________<br>77. Refused to answer/Alikataa kujibu<br>88. Does not know/ Hajui ☐
**5. What is your current occupation?/Unafanya kazi gani kwa sasa?**<br>1. Homemaker/Housewife/mama wa nyumbani<br>2. Employee/mwajiriwa<br>3. Self employment/nimejiajiri<br>4. Farming/mkulima<br>5. Jobless/sina kazi<br>6. Other/Nyingine______________<br>77. Refused to answer/Alikataa kujibu<br>88. Does not know/ Hajui ☐	**6. Have you suffered from any illnesses such as malaria or asthma within the past month?/Umeshawahi kuugua ugojwa wowote kwa kipindi cha mwezi mmoja uliopita?**<br>1. No/hapana<br>2. Yes, describe/Ndio, Fafanua_________<br>77. Refused to answer/Alikataa kujibu<br>88. Does not know/ Hajui<br>☐
**7. Have you suffered from any physical disability or restriction of movement within the last month?/Je umepata matatizo ya kimwili, au kuzuiwa kusogea kwa kipindi cha mwezi mmoja uliopita?**<br>1. No/hapana<br>2. Yes, Unable/difficulty walking/ndio,siwezi kutembea/natembea kwa shida<br>3. Yes, Limited use of arms/hands/ndio,siwezi kutumia mikono na viganja<br>4. Yes, Poor vision/ndio, sioni vizuri<br>5. Yes, other/Ndiyo,Nyingine_________________<br>77. Refused to answer/Alikataa kujibu<br>88. Does not know/ Hajui ☐	**8. Does this household own any working means of transport?/Una usafiri wowote unaomiliki?**<br>1. Yes, car / nidyo gari<br>2. Yes, Bicycle / Ndiyo baiskeli<br>3. Yes, Motorbike / Ndiyo pikipiki<br>4. Yes, other /ndiyo, nyinginezo ____________________<br>5. No / hapana<br>77. Refused to answer/Alikataa kujibu<br>88. Does not know/ Hajui<br>☐
**9. Do you have difficulty meeting your daily needs (e.g food, clothing, shelter, medicine)?/Je unapata shida kupata mahitaji yako ya kila siku (m.f chakula, dawa, nguo, malazi?)**<br>1. Yes/ndiyo<br>2. No/hapana<br>77. Refused to answer/Alikataa kujibu<br>88. Does not know/ Hajui<br>☐	**10. During the last month have you used any of the following?/Ndani ya mwezi uliopita, umetumia chcote kati ya vifuatavyo? (Circle all that apply / Zungushia zinazo husika)**<br>1. Seatbelt/mkanda wa kiti cha gari<br>2. Bicycle helmet/kofia ya baiskeli<br>3. Careful driving/kuendesha kwa uangalifu<br>4. None of the above / hakuna jibu<br>77. Refused to answer -/- Amekataa kujibu<br>88. Does not know answer -/- Hajui majibu<br>☐

B. Injury Event Form (Fomu ya tukio la jeraha)
Complete the below section for each child in the household whether they have experienced an injury within the last month. A separate form should be completed for each child.

Now we will ask you some questions about injuries that the children living in your household may have experienced during the last month. /*Tutakuuliza maswali kuhusiana na majeraha ambayo watoto wanaoishi kwenye nyumba yako waliwahi kuyapata kwa kipindi cha mwezi mmoja uliopita.*

<table>
<tr>
<td>1. How many children under 18 are under your care and live in the household? /watoto wangapi chini ya miaka 18 unawalea_________→
☐</td>
<td>→ <u>Interviewer</u>: select the first child starting with the eldest. (I would now like to ask you some questions about your children, starting with the oldest/nitakuuliza maswali kuhusiana na watotot wako tukianza na wa kubwa).
☐</td>
</tr>
<tr>
<td>2. Age of child (years)/ Umri wa mtoto
1. <1 year/miaka
2. 1-4
3. 5-9
4. 10-14
5. 15-18
77. Refused to answer/Alikataa kujibu
88. Does not know/ Hajui ☐</td>
<td>3. Gender of child / Jinsia ya mtoto
1. Male/Kiume
2. Female/Kike
☐</td>
</tr>
<tr>
<td>4a. Has this child had an injury within the last month? /Je, mtoto huyu amepata jeraha ndani ya mwezi ulio pita?
1. Yes/Ndiyo (go to question -------> Q5) /Hapana (nenda swali la 5)
2. No/Hapana (go to question -------> 20) / Kama hapana, ruka hadi swali la ishirini na mbili (20)
☐</td>
<td rowspan="2">5. Where was the child when the injury happened?/Kama ndiyo, mtoto alikuwa wapi wakati anaumia?
1. Own home inside / Nyumbani kwao ndani
2. Own home outside / Nyumbani kwao nje
3. Other home inside / Nyumbani kwa wengine ndani
4. Other home outside / Nyumbani kwa wengine nje
5. Road/street/highway / Barabarani, Mtaani, njia kuu
6. Farm, excluding home / shambani, kasoro Nyumbani
7. Market/Shopping center / sokoni
8. Industrial/Construction area / Viwandani/ sehemu inayo Jengwa
9. School/Education area / Shule/ Sehemu za kusomea
10. Other public building / Majengo mingine ya umma
11. Sports and play area / Michezo na sehemu za kuchezea
12. Countryside (water, sea) / sehemu zilizo na maji kama bahari
13. Other __________ / sehemu zingine
14. Refused to answer / Amekataa kujibu
15. Does not know answer / Hajui jibu ☐</td>
</tr>
<tr>
<td>4b. <u>Interviewer:</u> Mark this box if the injury resulted in a death
☐
☐</td>
</tr>
<tr>
<td>6a. What area of the child's body was injured? -/- Ni sehemu gani ya mwili ya mtoto iliyoumia?
1. Internal organs -/- Viungo vya ndani
2. Arms -/- Mikono
3. Legs -/- Miguu
4. Back -/- Mgongo
5. Chest -/- Kifua
6. Eyes -/- Macho
7. Hands -/- Mikono
8. Face -/- Uso
9. Mouth -/- Mdomo
10. Skin-/-ngozi
11. Multiple / Zaidi ya moja
12. Other/nyingine____________________
77. Refused to answer -/- Amekataa kujibu
88. Does not know answer -/- Hajui jibu
☐</td>
<td>7. Who initially tried to help the child? -/- Nani alijaribu Kumsaidia mtoto huyo awali
1. Myself / Mimi mwenyewe
2. Bystander -/- Mtu aliyekuwa amesimama karibu/mtazamaji
3. Friend/family -/- Rafiki/ Familia
4. Police -/- Polisi
5. Ambulance -/- Gari la wagonjwa mahututi
6. No help -/- Hakusaidiwa
7. Other/ Zingine________________
77. Refused to answer -/- Amekataa kujibu
88. Does not know answer -/- Hajui jibu
☐</td>
</tr>
</table>

**6b. Did the child break or fracture any bones?**<br>1. Yes, broken bone / Ndiyo alikunjika mfupa<br>2. Yes, fracture / Ndiyo alikunjika mfupa<br>3. No / hapana<br>77. Refused to answer -/- Amekataa kujibu<br>88. Does not know answer -/- Hajui jibu<br>☐	
**8. What kind of activity was the child doing at the time of injury?/Mtoto huyo alikuwa anafanya kitu gani wakati alipopata majeraha?**<br>1. Organized sports / Michezo<br>2. Leisure / Play /Mapumziko/ Kucheza<br>3. Paid work (including traveling) /Kazi ya kulipwa (ikiwepo kusafiri)<br>4. Unpaid work /kazi isiyo kuwa ya kulipwa<br>5. Educational activity /Mambo ya kimasomo<br>6. Activity of daily living (i.e. Cooking or Bathing including walking)/Mambo yanayo husu maisha ya kila siku (kama kupika au kuoga Pamoja na kutembea)<br>7. Other/nyingine _________<br>77. Refused to answer /Alikataa kujibu<br>88. Does not know answer /Hajui jibu<br><br>Go to question -------> Q9) /Nenda swali la 9 ☐	**9. What kind of injury did the child have?/ aina gani ya majeraha mtoto alipata?**<br>1. Burn/kuungua →Nenda swali la 10<br>2. Fall/kuanguka → Nenda swali la 11<br>3. Poisoning / sumu→ Nenda swali la 12<br>4. Animal/insect bite /kung'atwa na mnyama au mdudu→ Nenda swali la 13<br>5. Intentional injury/aliumizwa kwa kusudi → Nenda swali la 14<br>6. Motor vehicle collision /ajali ya gari→ Nenda swali la 15<br>7. Cut / Kujikata → Nenda swali la 17<br>8. Other/mengine__________→ Nenda swali la 17<br>77. Refused to answer /Alikataa kujibu 20<br>88. Does not know /Hajui jibu → Nenda swali la 20<br>☐
**10. What was the cause of the child's burn? /Nini kilisababisha mtoto huyu kuungua?**<br>1. Open Fire/Flame -/- Moto<br>2. Hot object -/- Chombo cha moto<br>3. Hot liquid -/- kitu cha moto cha majimaji<br>4. Steam /Mionzi ya maji moto<br>5. Smoke inhalation -/- kuvuta moshi<br>6. Chemical -/- Kemikali<br>7. Electric -/- Umeme<br>8. Other/nyingine _________<br>77. Refused to answer -/- Amekataa kujibu<br>88. Does not know answer -/- Hajui jibu<br>☐	**11. Where/what did the child fall from?/Mtoto huyu alianguka kutoka wapi?**<br>1. From bed, sofa, furniture /Kutoka kitandani, kochi, vifaa vya nyumbani kama meza<br>2. Down Stairs/Steps/Ladder -/- katika ngazi za nyumba<br>3. From attendants arms /Kutoka mikononi mwa aliye kuwa naye<br>4. Off Playground equipment /Kutoka katika vyombo vya kuchezea<br>5. Off rooftops/Kutoka juu ya paa la numba<br>6. Out of windows/ off balconies -/- Nje ya dirisha<br>7. From a tree / Kutoka juu ya mti<br>8. Other/nyingine _________<br>77. Refused to answer /Amekataa kujibu<br>88. Does not know answer /Hajui jibu.<br><br>Go to → question 17 ☐
**12. What type of poisoning was it? -/- Ilikuwa ni sumu ya aina gani?**<br>1. Medicine/ Pharmaceutical -/- Dawa<br>2. Kerosene or Paraffin -/- Mafuta taa<br>3. Fertilizer or insecticide -/- mbolea au dawa ya wadudu<br>4. Household cleaning agents -/- Madawa ya kusafisha nyumbani<br>5. Other/nyingine _________<br>77. Refused to answer -/- Amekataa Kujibu<br>88. Does not know answer -/- Hajui jibu<br><br>Go to → question 17 ☐	**13. What was the child bitten/stung by?/mtoto aling'atwa/dungwa na nini?**<br>1. Snake/nyoka<br>2. Bee/wasp/nyuki/dondola<br>3. Dog/mbwa<br>4. Cow/goat/ng'ombe/mbuzi<br>5. Other animal/wanyama wengine_________<br><br>Go to → question 17 ☐

**14. What was the intentional injury due to? / Jeraha la kukusudia ilisababishwa na nini?**<br>1. Stab with sharp object by another person / kuchonwa na ncha kali na mtu mwingine<br>2. Strike with blunt object by another person /kupigwa na kitu bapa na mtu mwingine<br>3. Strike with closed hand by another person / kupigwa na ngumi na mtu mwingine<br>4. Strike with open hand by another person / kupigwa kibao<br>5. Self-inflicted injury / majaraha yakujisababishia mwenyewe<br>12. Other /Zingine___________________________<br>77. Refused to answer /Amekataa kujibu<br>88. Does not know answer / Hajui jibu<br><br>Go to → question 17 ☐	**15. What type of vehicle was the child in during the collision? /alitumia njia ipi ya usafiri?**<br>1. Inside a passenger car /Sedan/Gari la abiria/gari ndogo<br>2. 3 wheeler/Bajaji<br>3. Daladala<br>4. Pickup/LDV/van/jeep(< 10 people)/gari ndogo ya mizigo (pikapu), jipu, vani(< watu 10)<br>5. Truck/Heavy vehicle -/-Lori/ Gari kubwa<br>6. Train /Treni<br>7. Motorcycle/moped -/-Pikipiki /baiskeli moto<br>8. Animal or animal drawn vehicle /myama au vyombo vinavyo vutwa na wanyama<br>9. Bicycle /Baisikeli<br>10. Boat /Boti<br>11. Walking/kutembea<br>12. Other/Zingine__________________<br>77. Refused to answer /Amekataa kujibu<br>88. Does not know answer / Hajui jibu ☐
**16. What was the striking vehicle or object?/Ni gari lipi au chombo kipi kilicho mgonga?**<br>1. Passenger car /Gari la abiria<br>2. 3 wheeler /Bajaji/ Rickshaw<br>3. Bus/Van-/-Basi/ Vani/ Daladala<br>4. Truck/Heavy vehicle -/-Lori/ Gari kubwa<br>5. Train/Treni<br>6. Motorcycle/Pikipiki<br>7. Horse /Farasi<br>8. Bicycle /Baisikeli<br>9. Boat /Boti<br>10. Animal or animal-drawn /mnyama au vyombo vinavyo vutwa na wanyama.<br>11. Fixed or stationary object /chombo kilichotulia au kisicho weza kuondolewa.<br>12. Other/nyingine __________<br>77. Refused to answer/Amekataa kujibu<br>88. Does not know answer /Hajui jibu ☐	**17. Have there been any other injuries to this child within the past month? -/- Je, mtoto huyu amepata majeraha mengine ndani ya mwezi ulio pita? (circle all that apply/ Zungushia zinazo husika)**<br>1. No (go to question → Q18)/ hapana, nenda swali la 18<br>2. Vehicle collision/kugongwa na gari<br>3. Near drowning/ drowning -/- Anakaribia kubebwa na maji/ alibebwa na maji<br>4. Cut -/- Kujikata<br>5. Struck/hit by object -/- Amekwama/ kagogwa na chombo<br>6. Choking on food -/- Amepaliwa na chakula<br>7. Entrapment in closed space -/- Ameshikwa sehemu iliyo fungwa<br>8. Dog bite -/- kung'atwa na mbwa<br>9. Snake bite -/- Kung'atwa na nyoka<br>10. Other animal bite -/- Kung'atwa na myama mwingine<br>11. Exposure to excessive heat -/- Kuwa katika sehemu ya joto sana<br>12. Machinery injury -/- Majeraha ya mashine<br>13. Other/nyingine ______________<br>77. Refused to answer -/- Amekataa kujibu<br>88. Does not know answer -/- Hajui Jibu ☐
**18. What do you think will be the long-term effect of this injury? -/- je, unakadiria majeraha haya yatamdhuru vipi kwa maisha ya mbeleni?**<br>1. No significant disability -/- Hamna dalili zozote za ulemavu<br>2. Short term temporary disability (less than 6 weeks) -/- Ulemavu wa muda mfupi (chini ya wiki 6)<br>3. Long term temporary disability (6 or more weeks) -/- Ulemavu wa siku nyingi (wiki 6 na zaidi)<br>4. Permanent disability -/- Ulemavu usiyo isha/ wa milele<br>5. Death / kifo<br>6. Other/zingine_________________________<br>77. Refused to answer -/- Amekataa kujibu<br>88. Does not know answer -/- Hajui Jibu ☐	**19. Did the family have to borrow money to care for the child? -/- Je, ilibidi familia ikope hela ili waweze kumuhudumia mtoto?**<br>1. Yes -/- Ndiyo<br>2. No -/- Hapana<br>77. Refused to answer -/- Amekataa kujibu<br>88. Does not know answer -/- Hajui jibu?<br><br>Go to question→ Q21) /Hapana (nenda swali la 21) ☐

**20. If the child was injured, where would you first go for help in case of an injury? -/- Kama mtoto wako angeumia, je, ungeenda sehemu ipi ya kwanza kupata usaidizi?**<br>1. Hospital -/- Hospitalini<br>2. Community health worker -/- kutoka kwa mfanya kazi wa afya ya jamii<br>3. Drug store -/- kutoka kwa duka la dawa baridi<br>4. Traditional doctor -/- kutoka kwa mganga wa kienyeji<br>5. Police/polisi<br>6. Neighbor/jirani<br>7. Other/nyingine_______________<br>77. Refused to answer -/- Amekataa kujibu<br>88. Does not know answer -/- Amekataa kujibu ☐	**21. Has this child suffered from any illnesses within the past month such as malaria or asthma? -/- mtoto huyu aliugua ugonjwa sugu wowote kwa kipindi cha mwezi mmoja uliopita kama vile malaria?**<br>1. Yes, Describe/ ndiyo, fafanua______________<br>2. No/ hapana<br>77. Refused to answer -/- Amekataa kujibu<br>88. Does not know answer -/- Hajui majibu<br>☐

C. Household Description Form (Fomu ya Maelezo ya Kaya)
I'm going to end the survey by asking you some questions about your household. (Nitaanza utafiti kwa kukuuliza maswali kuhusu kaya yako)

**1. Is this household in a multi-level apartment building?/je nyumba hii ni ya ghorofa?**<br>1. Yes/Ndiyo<br>2. No/Hapana<br>☐	**2. How many rooms does your family use in this household? / Vyumba vingapi familia yako inatumia kwenye kaya hii?________**<br>77. Refused to answer/Alikataa kujibu<br>88. Does not know/ Hajui ☐
**3. Does the household have any of the following? (circle all that apply/Zungushia zinazo husika)/Je, muhusika ana chochote kati ya vifuatavyo?**<br>1. A Working television/ runinga inayo tumika<br>2. A Working radio/ Je, mnamiliki redio?<br>3. A working mobile phone/ Je, unayo simu ya mkononi?<br>4. All of the above/zote zilizo tajwa<br>5. None of the above/hamna kati ya zilizotajwa<br>77. Refused to answer/Alikataa kujibu<br>88. Does not know/ Hajui<br>☐	**4. What is the primary fuel used for cooking in the household?/je hapa nyumbani mnatumia nishati gani kwa kupikia?**<br>1. Electricity/umeme<br>2. Coal/mkaa<br>3. Gas/gesi<br>4. Wood/kuni<br>5. Kerosene / Mafuta ya taa<br>6. Dung/samadi<br>7. Other/nyinginezo________<br>77. Refused to answer/Alikataa kujibu<br>88. Does not know/ Hajui ☐
**5. How many people sleep overnight in this household? /je watu wangapi wanalala katika nyumba hii?______________**<br>77. Refused to answer/Alikataa kujibu<br>88. Does not know/ Hajui ☐	
